# Seed Coating with Hydro-Absorbers as Potential Mitigation of Early Season Drought in Sorghum (*Sorghum bicolor* L. Moench)

**DOI:** 10.3390/biology6030033

**Published:** 2017-07-31

**Authors:** Linda Gorim, Folkard Asch

**Affiliations:** 1 51 Campus Drive Saskatoon, Department of Plant Sciences College of Agriculture and Bioresources, University of Saskatchewan, SK S7N5A8, Canada; linda.gorim@usask.ca; 2 Institute for Agricultural Sciences in the Tropics (Hans-Ruthenberg-Institute) Garbenstr. 13, University of Hohenheim, 70599 Stuttgart, Germany

**Keywords:** Susu, Piper, root-shoot-ratio, Mantelsaat, leaf development

## Abstract

Climate change poses a threat to sorghum production systems by shifting the onset of the rainy season to a later date, increasing the risk of crop failure during crop establishment. The effects of drought on sorghum during seedling establishment have not been determined. Coating seeds with a water absorbing substance offers a way to buffer the seed against insufficient moisture in the surrounding soil. Seeds of two different sorghum varieties were coated with one of two commercially available hydro-absorbers: Stokosorb^®^ and Geohumus^®^. These hydro-absorbers have the capacity to store water several times their own weight. The aim of this study was to compare the effects of the cited hydro-absorbers on early seedling growth of two sorghum landraces under different levels of soil water deficit. Seedlings were grown for 12 days under three water availability levels (Field capacity (FC), 50% of FC, and 25% of FC). The seedlings under water limited treatments were subsequently re-watered. Biomass, root length, plant height, leaf area, and leaf extension rate were monitored in two-day intervals for 24 days. Coating strongly affected seedling growth both under fully watered and water deficit conditions. Sorghum varieties differed in their responses to both soil water deficit and coating materials. In general, Stockosorb improved seedling performance under water limited conditions particularly by promoting root growth, whereas Geohumus did not.

## 1. Introduction

Sorghum is one of the most important staple food crops in Africa, particularly in the drier and more marginal areas of the semi-arid tropics of Africa [1,2]. It is grown as a rain-fed crop, and is sown after the first rains of the rainy season. Climate change threatens sorghum production systems by not only delaying the onset of the rainy season [3], but also increasing the variability in time between the first and the second rains, leading to a greater risk of crop failure during crop establishment [4,5]. Sorghum has been reported to be susceptible to variable soil moisture content during germination [6,7] but highly resistant to drought stress at the seedling stage [8]. Whereas sorghum drought tolerance traits and characteristics have been intensively studied in the late vegetative stage, at flowering and for grain yield (e.g., [9,10,11,12,13]), the effects of drought on sorghum during the critical period of seedling establishment have not yet been determined. In order to mitigate the potentially detrimental effects of variable rainfall during seedling establishment, it is important to improve understanding of sorghum seedling responses to dehydration between germination and crop establishment. One possibility that could improve seedling resistance to drought is to develop options to improve the supply of water to seedlings during the establishment phase. Insufficient soil moisture can be buffered by coating seeds with a water absorbing substance. Seed coating technology has a variety of applications, such as delivering nutrients [14,15], peroxides to provide oxygen, hormones to improve growth [16] or hydro-absorbers to improve water supply [17,18]. Geohumus and Stockosorb are two commercially available hydro-absorbers able to store an amount of water several times their weight. Seeds of two contrasting sorghum varieties were coated with either of the hydro-absorbers via the Mantelsaat technology of Freudenberger Feldsaaten GmbH. The aim of this study was to compare the effects of the hydro-absorbers contained in the coat on early seedling growth under different levels of drought stress. Takele [7] proposed using a seedling’s shoot dry weight, root length, and leaf area to screen for drought tolerance in sorghum. In this study, we investigated the proposed parameters for the two varieties with coated and uncoated seeds at different levels of soil moisture deficit. The main hypotheses were: (a) seedlings grown from grains coated with the hydro-absorber will have higher seedling survival rates and improved growth after re-watering following a drought spell, due to the greater amount of moisture available during the critical period of seedling establishment; and (b) the different sorghum varieties will respond differently to both drought stress and hydro-absorber coatings.

## 2. Materials and Methods

### 2.1. Plant Materials and Growth Conditions

Seeds of two sorghum varieties (cv. Susu and cv. Piper) were obtained from Freudenberger Feldsaaten GmbH and used in all experiments. Treatments for each variety comprised: (i) uncoated seeds; (ii) seeds coated with Stockosorb; and (iii) seeds coated with Geohumus. Both hydro-absorber containing coats were developed by Freudenberger Feldsaaten GmbH. The experiments were carried out in a greenhouse at the University of Hohenheim, Stuttgart Germany, in July–August for two consecutive years. Plants were illuminated for 12 hours daily with sodium vapor lamps resulting in a mean light intensity of about 600 µmol m^−2^ s^−1^. The mean temperature and relative humidity were logged with TinyTags (Gemini Data Loggers Inc, Chichester, United Kindom) and ranged from 26.5 ± 4.3 °C to 28.2 ± 5.4 °C and 48.4 ± 13.9% to 53.2 ± 17.6%, respectively, in the first season, and 24.3 ± 5.1 °C to 27.9 ± 6.5 °C and 48.6 ± 17.2% to 53.7 ± 16.2% in the second season. 

### 2.2. The Mass of Sorghum Grains

The average mass of the uncoated seed was 22.5 (± 3.9) mg and 9.9 (± 1.1) mg for Susu and Piper, respectively. Grains comprised of a seed and a coating containing Stockosorb (hereafter referred to as Stockosorb Grain) had an average mass of 65.9 (± 13.5) mg and 35.4 (± 2.4) mg, whereas grains comprised of a seed and a coating containing Geohumus (hereafter referred to as Geohumus Grain) had an average mass of 71.6 (± 23.0) mg and 37.1 (± 11.6) mg for Susu and Piper, respectively.

### 2.3. Experimental Setup

**Figure 1:**
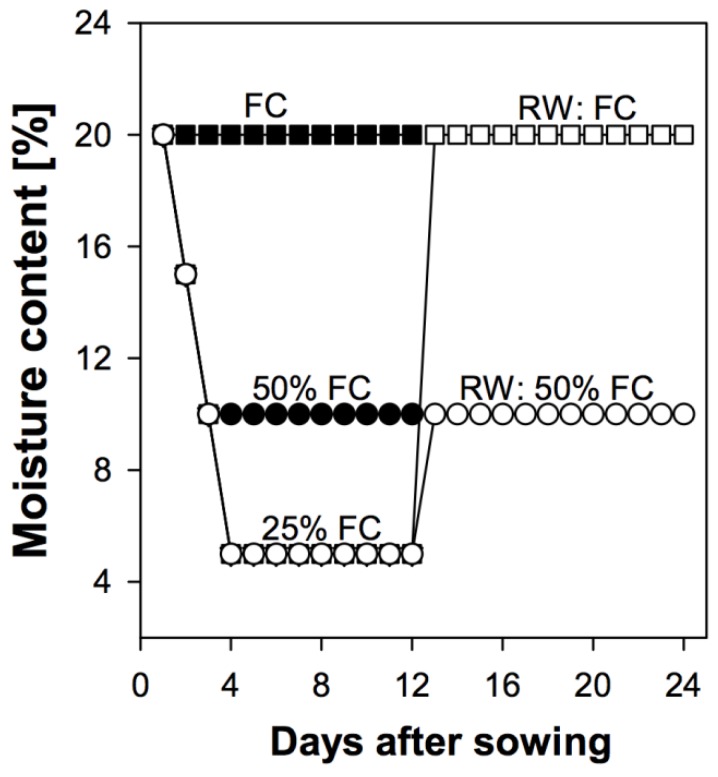
Kinetics of soil moisture content during drought spell and re-watering of sorghum seedlings to 50% FC and FC, where FC denotes the field capacity and RW denoted re-watering.

Pots (11 × 11 × 20 cm) filled with medium coarse sand (2 kg), to which 60 mL Clark nutrient solution [19] (diluted 1:5) was added, were used in all experiments. Each pot contained seedlings (number per pot depending on the experiment) of each variety and coating type. For each combination, 3 replicates were established under fully watered conditions, termed field capacity (FC = 20% gravimetric moisture content) and at 2 moisture deficit levels: 50% FC (moderate water deficit) and 25% FC (severe water deficit). Treatments were maintained for 12 days, then re-watered according to Figure 1 and kept at the new moisture levels for another 12 days. Resulting gravimetric moisture contents of the different treatments are shown in Figure 1. All pots were maintained at these moisture levels by measuring twice daily the difference in weight and adding an appropriate amount of water.

#### 2.3.1. Determination of Early Seedling Root Length and Dry Matter

Every other day from the fourth day after sowing (DAS), 3 pots of seedlings from all three seed treatments were randomly selected, destructively harvested, and soil was washed from the roots. The root length of each harvested seedling was measured with a ruler. Seedlings were separated into root and shoot and oven-dried at 70 °C to constant weight. The dried samples were weighed using a fine balance and root/shoot ratio and total biomass determined. 

#### 2.3.2. Determination of Plant Height and Leaf Parameters

The experimental design for each pot: 8 seeds were planted, 2 at 4 spots, 7 × 7 cm apart and later thinned to one at each spot. The plant height from soil level to the tallest leaf tip was determined at 10 DAS for both varieties. The leaf length (from base to tip) and widest width were measured daily with a ruler for the second and third leaves for both varieties from 4 DAS onwards, allowing for non-destructive determination of leaf elongation rate and leaf area [20]. The leaf area of 5 random leaves from each treatment was determined with the leaf area meter (MK2, Delta-T, England) and their length (L) and width (W) with a ruler in other to calculate the corrections factor (F) required for total leaf area estimation. The leaf area (y) was calculated from the formula: y = L × W × F. The leaf width measurements began when at least 80% of the leaf had unfolded. The lengths of the second, third, and in some cases fourth leaves of seedlings depending on variety were measured. Leaf elongation rate (LER) was thus calculated as the change in leaf length between any given day and that at 4 DAS, divided by the time in between measurements in days.

#### 2.3.3. Seedling Growth after Re-Watering 

Susu seedlings grown at 25% FC were re-watered at 12 DAS to either field capacity or 50% of field capacity (Figure 1). The root length and total biomass were assessed over a 12-day period as described above. Susu was chosen for re-watering because drought effects on its seedlings were significantly more severe than in Piper. 

#### 2.3.4. Statistical Analysis

Least significant differences were calculated with an analysis of variance at 5% alpha using the LSmeans and means statement in the general linear model (GLM) procedure of SAS. Graphs were drawn with Sigma Plot 10, Systat Software Inc., Germany.

## 3. Results

### 3.1. Coating Effects on Seedling Growth under Fully Watered Conditions

**Figure 2:**
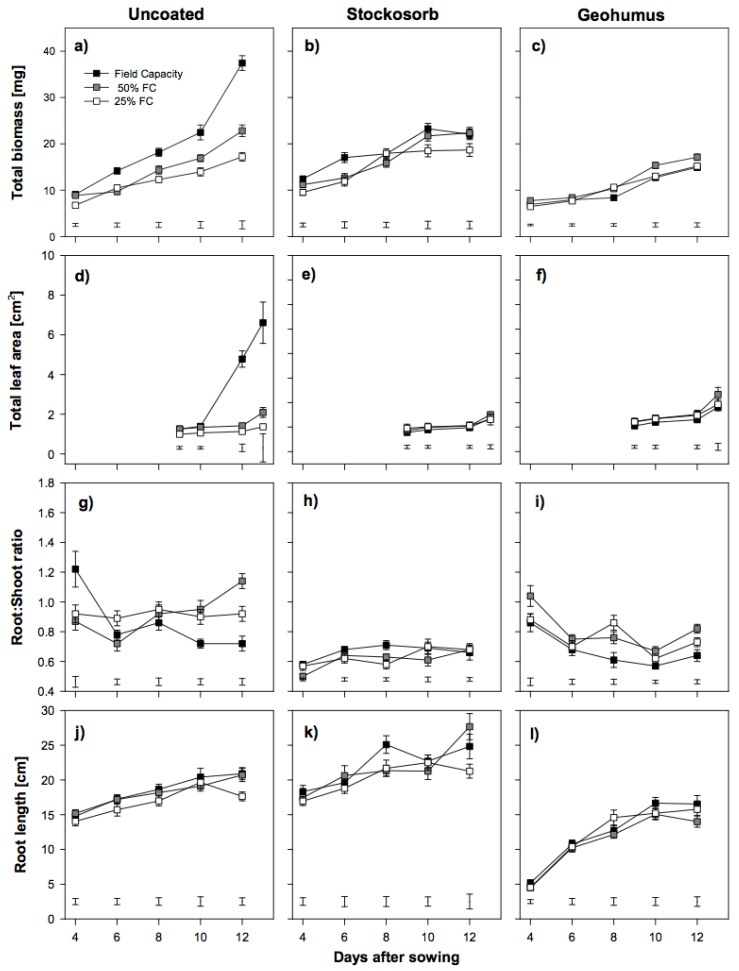
Effects of drought stress on total biomass, leaf area, root:shoot ratio and root length of Piper uncoated (**a**, **d**, **g**, **j**) coated with two hydro-absorbers, Stockosorb (**b**, **e**, **h**, **k**) and Geohumus (**c**, **f**, **i**, **l**). (Bars on data points denote standard error while those in graphs indicate the least significant difference at α = 5% and FC = field capacity).

Coating sorghum seeds with either hydro-absorber affected the growth of seedlings of both varieties, independent of the degree of soil moisture deficit. Under fully watered conditions, Piper seedlings grown from both Stockosorb and Geohumus grains showed reduced total biomass, LA and root:shoot ratio (RSR) compared to those grown from uncoated seeds (Figure 2). 

**Figure 3:**
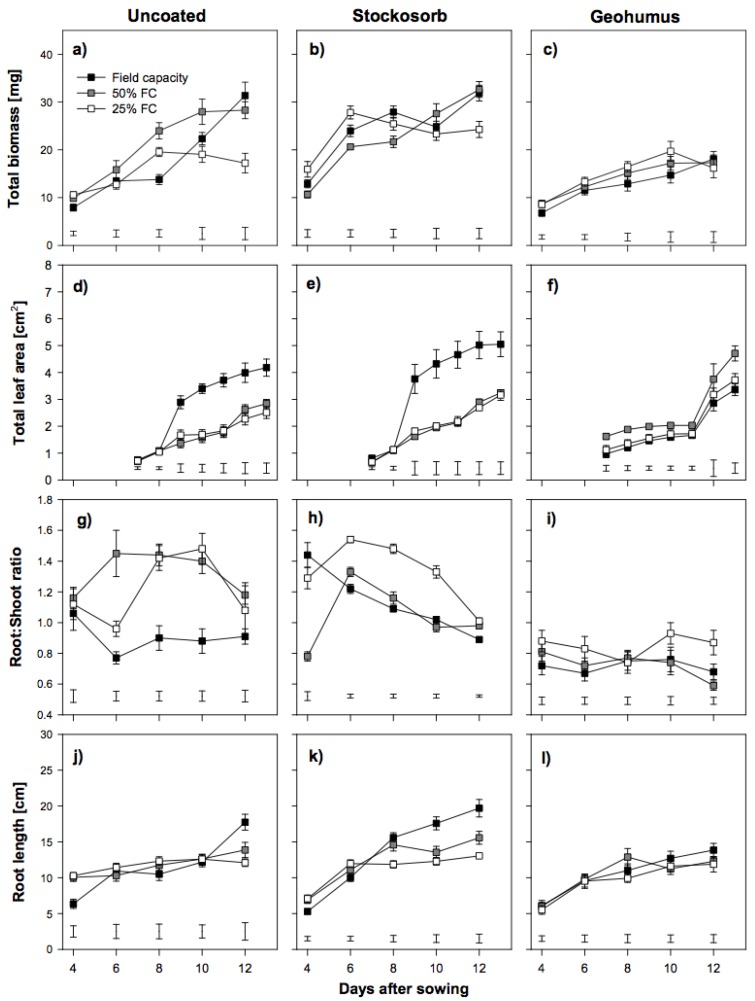
Effects of drought stress on total biomass, leaf area, root:shoot ratio and root length of Susu seedlings uncoated (**a**, **d**, **g**, **j**) coated with two hydro-absorbers, Stockosorb (**b**, **e**, **h**, **k**) and Geohumus (**c**, **f**, **i**, **l**). (Bars on data points denote standard error while those in graphs indicate the least significant difference at alpha = 5% and FC = field capacity).

Piper variety seedlings grown from Stockosorb grains had slightly increased root length, whereas those grown from Geohumus grains had significantly reduced root length, especially in comparison to the seedlings grown from uncoated seeds (Figure 2j–l). In contrast, Susu seedlings grown from Stockosorb grains under fully watered conditions did not have significantly reduced biomass or RSR and promoted root growth. These parameters were reduced in Susu seedlings grown from Geohumus grains (Figure 3). 

**Figure 4:**
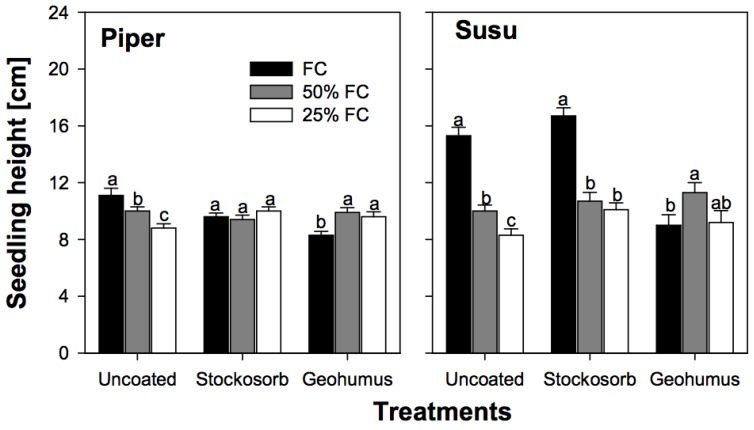
A comparison of the effect of drought stress on the height of coated and uncoated Piper and Susu seedlings assessed on the tenth day after sowing. (FC denotes soil moisture content at field capacity, bars indicated standard error of the mean and letters on bars are a comparison between the different moisture levels for the same treatment).

Piper seedlings grown from either hydro-absorber grains were significantly shorter under fully watered conditions compared to those from uncoated seeds. In contrast, the height of Susu seedlings grown from Stockosorb coated seeds was unaffected, whereas height under fully watered conditions in seedlings grown from Geohumus grain was significantly reduced (Figure 4). 

In Susu seedlings, the leaf elongation rate (LER) of the second leaf was significantly higher for seedlings grown from Geohumus grains, whereas those grown from either Stockosorb grains or uncoated seeds had similar LER under fully watered conditions (Table 1). However, the LER of the third leaf in Susu seedlings was similar in seedlings grown from Stockosorb grains and uncoated seeds and significantly higher at α = 5% than the LER of seedlings grown from Geohumus grains (Table 1). In Piper seedlings, LER of the second leaf was significantly higher for seedlings grown from uncoated seeds, when compared to those grown from coated seeds. However, Piper seedlings grown from Geohumus coated seeds had a higher LER in the second leaf compared to their counterparts grown from Stockosorb coated seeds (Table 1). A similar trend was observed in the LER of the third leaf of Piper seedlings under fully watered conditions, but seedlings grown from Stockosorb grains showed a significantly higher LER compared to their counterparts grown from Geohumus grains (Table 1).

### 3.2. Seedling Growth under Water Deficit without Hydro-Absorber Coatings

Water availability strongly influenced early seedling growth in both varieties. However, each variety’s response to moisture deficit varied. Susu seedlings had greater biomass than Piper seedlings. Piper seedlings grown under fully watered conditions produced the largest biomass, but when under severe water deficit produced the smallest (Figure 2a). The greatest biomass was observed in Susu seedlings grown from uncoated seeds at 50% FC (Figure 3a). However, total biomass was reduced by more than 60% in Piper and about 50% in Susu seedlings grown from uncoated seeds under severe water deficit (Figure 2a and Figure 3a). This was reflected in the leaf area (LA) (Figure 2d and Figure 3d). LA was reduced by 75% in Piper seedlings and 50% in Susu seedlings grown from uncoated seeds under severe water deficit. In fully watered conditions, Susu seedlings accumulated only about one third of the total LA produced by Piper seedlings also grown from uncoated seeds (Figure 2d and Figure 3d). Water deficit resulted in an increase in the RSR for both varieties. In Piper seedlings, the RSR was on average 20% higher under both water deficit treatments, whereas, in Susu seedlings, the average increase was 50% (Figure 2g and Figure 3g). In both varieties, root length was unaffected by water deficit. Susu seedlings had significantly longer roots (Figure 2j and Figure 3j). Whereas water deficit had no effect on seedling height in Piper seedlings, it was reduced by 40% and 50% under moderate and severe water deficit respectively in Susu seedlings (Figure 4). In Susu seedlings under severe water deficit, LER was reduced by 23–21% for the second and 53–40% for the third leaf, whereas, in Piper seedlings, LER was reduced by 9–24% for the second and 53–57% for the third leaf (Table 1).

### 3.3. Effect of Coating on Seedling Growth during Water Deficit

Coating the seeds of the two sorghum varieties with hydro-absorbers negatively affected seedling performance under fully watered conditions and did not produce the expected positive results under water deficit conditions. Piper seedlings grown from Stockosorb and Geohumus grains had significantly lower biomass than those from uncoated seeds irrespective of the moisture level (Figure 2a,b). Under water deficit conditions, the total LA in Piper seedlings grown from coated or uncoated seeds was similar (Figure 2e,f). The RSR was lowest in seedlings grown from Stockosorb grains under both moderate and severe water deficit (Figure 2h) compared to seedlings grown from Geohumus grains and uncoated seeds. However, Piper seedlings grown from Stockosorb grains had significantly longer roots compared to any other seedling (Figure 2j–l). Susu seedlings grown from Stockosorb grains produced more biomass under severe water deficit compared to seedlings grown from uncoated seeds and at all moisture levels, the amount of biomass produced by these seedlings was higher than in seedling grown from Geohumus grains (Figure 3b,c). The total LA under water deficit was similar between seedlings grown from Stockosorb grains and uncoated seeds; however, for seedlings grown from Geohumus grains under water deficit, there was a significant increase from 10 DAS (Figure 3d–f). The RSR of seedlings grown from Stockosorb grains was significantly increased compared to all other treatments, while the RSR of seedlings from Geohumus grains was lower irrespective of the moisture level (Figure 3g–i). Root lengths were similar under water limitations between treatments although seedlings grown from Geohumus grains had shorter roots (Figure 3j–l). Under water deficit, Piper seedlings grown from Stockosorb grains had similar heights to fully watered seedlings, whereas in Susu, the height of seedlings growing under water deficit was significantly reduced at α = 5% (Figure 4). Seedlings grown from Geohumus grains of both varieties were significantly taller at α = 5% under water deficit than under fully watered conditions (Figure 4). Under water deficit conditions, the LER of seedlings from both Geohumus and Stockosorb grains was significantly higher than those from uncoated seeds, and higher in seedlings grown from Geohumus grains than those from Stockosorb grains (Table 1). In fully watered conditions, seedlings grown from Stockosorb grains showed a reduced LER (57%–31%) and (83%–30%) in the second and third leaves respectively. At nine DAS, the LER of the third leaf was significantly lower for seedlings grown from Geohumus grains under fully watered conditions. The LER of seedlings grown from both Geohumus and Stockosorb grains was similar under both moderate and severe water deficit (Table 1). 

### 3.4. Effect of Re-Watering on Seedlings Growth

**Figure 5:**
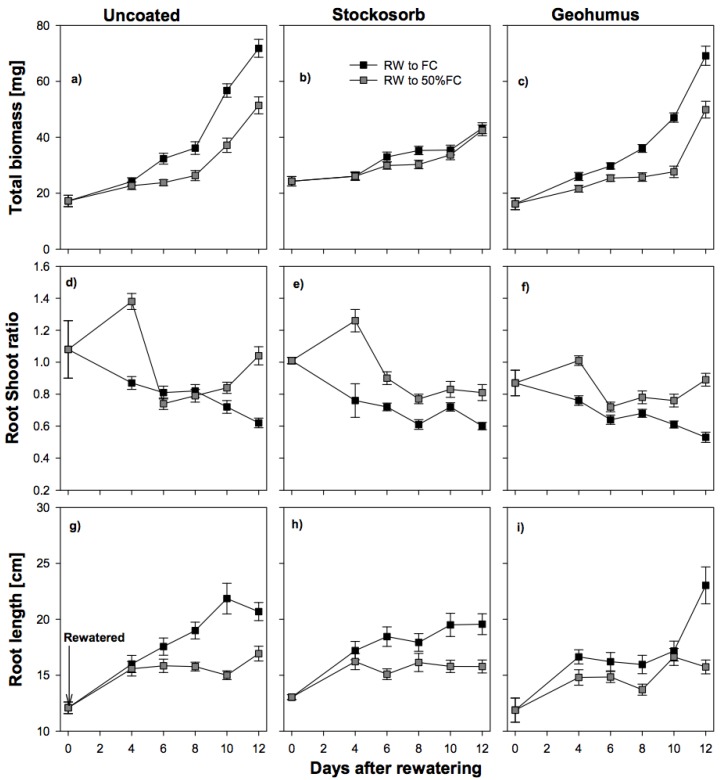
Response of root length, root:shoot ratio and total biomass of uncoated (**a**, **d**, **g**) and coated (**b**, **e**, **h** = Stockosorb; **c**, **f**, **i** = Geohumus) Susu seedlings to re-watering to field capacity and 50% field capacity. RW = re-watered. Error bars = standard error of means.

Susu seedlings grown under severe water deficit (25% FC) were re-watered to two moisture levels: FC and 50% FC. Root length, RSR, and total biomass were monitored for 12 days, day zero being the day that re-watering occurred. Irrespective of the level of watering, the highest biomass was observed in seedlings grown from uncoated seeds and Geohumus grains, but lower in seedlings grown from Stockosorb grains (Figure 5a–c). When re-watered to FC, the RSR decreased over time in seedlings grown from both coated and uncoated seeds but when re-watered to 50% FC, RSR was higher in seedlings grown from coated seeds (Figure 5d–f). Under fully watered conditions, seedlings grown from both coated and uncoated seeds had longer roots than those at 50% FC and uncoated seeds had the longest roots (Figure 5g–i).

## 4. Discussion

### 4.1. Effects of Coating on Seedling Growth without Water Limitations

Without water limitations, biomass accumulation was strongly reduced in seedlings of both varieties grown from Geohumus grains, but not to the same degree. In Piper seedlings grown from Stockosorb or Geohumus grains, biomass was lower than in those grown from uncoated seeds. This may be due to a coat-induced delay in the onset of germination as reported earlier by Gorim and Asch [18], poor leaf growth in seedlings from coated seeds (Figure 2e,f) and, in the case of seedlings grown from Geohumus grains, poor root development. In contrast, biomass accumulation was promoted in Susu seedlings grown from Stockosorb grains, but significantly reduced in seedlings grown from Geohumus grains as compared to uncoated seeds. Stockosorb is known to absorb water about 40 times its weight and therefore is expected to promote plant growth under water limited conditions. This was true for Susu but not Piper and we suggest this may be associated with genotypic susceptibility to water stress and could also be affected by the seed shapes and sizes. 

Similarly, coats containing Stockosorb or Geohumus strongly reduced leaf area development in Piper seedlings as compared to uncoated seeds (Figure 2d–f), whereas, in Susu, Stockosorb promoted early leaf growth and Geohumus strongly reduced it. The reason for this could be that the share of grain reserves mobilized for growth during germination was greater than for the uncoated seeds [21].

In addition, contrasting root development was observed between the varieties in their response to seed coating The RSR of Piper seedlings grown from Geohumus grains and uncoated seeds decreased over time, whereas RSR of seedlings grown from Stockosorb coated seeds remained constant. This was the reverse in Susu seedlings grown from Geohumus grains and uncoated seeds. They had a constant RSR, while RSR decreased in seedlings grown from Stockosorb grains. In both varieties, the longest roots were found in seedlings grown from Stockosorb grains, which agreed with Gorim and Asch [18], who showed that Stockosorb promoted root development. Seedlings grown from Geohumus grains showed poor root development due to the coating, which may act as a physical barrier and slow down root growth after germination.

### 4.2. Effects of Water Deficit on Seedlings Grown from Uncoated Seeds

Water deficit results in reduced seedling growth as a result of restricted cell division and enlargement [22]. Growth of above- and below-ground structures of seedlings of both varieties grown from uncoated seeds was negatively affected by water deficit. The total biomass produced by Piper seedlings grown under water deficit increased over time but to a lesser extent than seedlings grown under fully watered conditions (Figure 2a). This does not agree with reports suggesting sorghum seedlings produce higher dry matter under water stress [23]. Susu seedlings grown under 50% FC produced the largest total biomass compared to under fully watered conditions or severe water deficit. A possible explanation is that under fully watered conditions, the amount of water attracted by the coating led to over-saturation resulting in reduced seed metabolism. At 50% FC, over-saturation was avoided, and the partitioning of seed reserves towards growth was favored, as shown in barley [18].

Piper seedlings grown at 50% FC showed a significantly higher LA at 14 DAS compared to seedlings grown at 25% FC. This was reflected in the second and third leaf elongation at 50% FC than at 25% FC, a pattern that is in agreement with results reported by Kameli and Losel [24], who found reduced LA in stressed wheat seedlings. In Susu seedlings, the total LA under both water deficit conditions was similar but lower than under fully watered conditions. Furthermore, the second and third LER of these seedlings was similar, irrespective of water deficit, but lower in seedlings grown under fully watered conditions. This indicates reduced cell division and cell size resulting in lower LER and therefore, shorter plants as the result of moisture deficit (Figure 4), also observed in maize leaves under water stress [25].

In response to a deficit of moisture in the soil, plants reallocate resources to their roots, resulting in higher RSR. This pattern was observed in Piper seedlings grown at 50% FC. Seedlings grown at 25% FC showed a constant RSR, likely due to arrested growth. In Susu seedlings, a strong increase in RSR was observed in seedlings grown under both water deficit levels compared to those grown under fully watered conditions. In both varieties, there was no significant difference in root length between seedlings under water deficit conditions and fully watered conditions. This disagrees with reports from Gill et al. [23] and Bibi et al. [26], who found reduced root length in sorghum seedlings grown in PEG. However, the reduction in root length was shown not to be significant in all sorghum accessions [27]. Therefore, the observed differences in RSR in Susu between fully watered and water deficit conditions may have resulted from larger roots, whereas in Piper seedlings, roots were long but thin.

### 4.3. Effects of Soil Moisture Deficit on Seedlings Growing from Coated Seeds

Piper seedlings accumulated similar amounts of total biomass at 50% FC, either when grown from Stockosorb grains or from uncoated seeds. This implies that the Stockosorb coating reduced the direct effects of water deficit on the seedling, which improved the total biomass production. However, water stress has been reported to reduce the amount of biomass produced in sorghum accessions [26]. Although seedlings grown from Geohumus grains produced less biomass under water deficit conditions than seedlings grown from Stockosorb grains or uncoated seeds, biomass accumulation was either similar or higher than seedlings grown under fully watered conditions. This suggests that the coating mitigates some of the negative effects of water deficit.

Leaf growth is known to be very sensitive to changes in water potential [24]. In Piper seedlings under water deficit, the presence of hydro-absorbers in the coat did not significantly improve total LA (Figure 2d–f). In contrast, Susu seedlings grown under water stress, increased their total LA over time in seedlings grown from Stockosorb grains and uncoated seeds (Figure 3d,e).Seedlings grown from Geohumus grains at 50% FC had the highest LA compared to those grown at 25% FC (Figure 3f). The results support that the hydro-absorber coating attracted and preserved moisture to reduce the effects of water stress in seedlings. In water deficit conditions, coating seeds with Stockosorb did not significantly improve seedling height, but seedlings grown from Geohumus grains were taller (Figure 4). This is in agreement with results of Nha [27], who showed that Geohumus promoted leaf growth.

In Piper seedlings grown from Stockosorb grains, low RSR values were observed under water deficit, implying the Stockosorb coating attracted more water towards the seedling, allowing for greater shoot rather than root growth and hence a lower RSR. This was not observed in seedlings grown from Geohumus grains under water deficit, where higher RSR were found compared to seedlings grown from uncoated seeds. A similar result was observed under drought stress in maize plants when Geohumus was incorporated into the soil [22]. Susu seedlings grown from Stockosorb grains at 50% FC, had a lower RSR with a decreasing trend compared to seedlings grown from uncoated seeds, but RSR was still higher than the RSR in seedlings grown from Geohumus grains, which had the highest RSR at 25% FC but the shortest roots. This corresponded with reports by Bibi et al. [27] that sorghum accessions showed varied but reduced root lengths under water stress.

### 4.4. Interaction between Soil Moisture Deficit, Coatings and the Two Sorghum Varieties

Independent of the moisture level, seedlings of either sorghum variety grown from Geohumus grains produced lower biomass. Seedlings grown from Stockosorb grains performed best across varieties under severe water deficit, producing more biomass than the seedlings grown from uncoated seeds. Susu seedlings grown from Stockosorb grains produced the most biomass under all moisture levels (Figure 3a–c). The difference in responses of Piper and Susu may be due to the way starch reserves were mobilized into sugars in the presence of Stockosorb or Geohumus in the coating during seedling growth. It has been established that the presence or absence of oxygen plays a significant role in deciding which sugar pathway is followed [22,28,29]. In general, LA was significantly reduced in Piper seedlings compared to Susu, but the LER of seedlings grown from either Geohumus or Stockosorb grains was higher in Piper at all moisture levels. However, Piper seedlings grown from Geohumus grains had lower second and third LER under fully watered conditions.

RSR of the fully watered Susu seedlings grown from Stockosorb grains was lower than under severe water deficit, but the opposite was true for Piper. This could be attributed to genotypic differences. Root length growth was most strongly suppressed under water deficit in Piper seedlings grown from Geohumus grains. Stockosorb did not strongly improve root length in Susu, as observed in Piper.

### 4.5. Response of Seedlings to Re-Watering

By re-watering, we tried to simulate rainfall events (i.e., by re-watering back to field capacity) and rain showers (i.e., by re-watering back to 50% FC). The focus was on Susu and not Piper seedlings because Piper seedlings grown at 25% FC were severely affected by water deficit conditions, indicated by furled and tiny leaves. During the 12-day re-watering period Piper seedlings did not recover. Re-watered seedlings grown from coated seeds had lower total biomass, especially in seedlings grown from Stockosorb grains. Seedlings were unable to fully recover, supported by Xu et al. [30], who stated that grasses exposed to extreme drought produced lower biomass after re-watering. However, the amount of biomass produced by seedlings grown from Geohumus grains increased after re-watering while those from Stockosorb decreased. Over time, the different coatings had a significant effect on the RSR of seedlings re-watered to 50% FC, as these seedlings mobilized resources mainly to the roots. Re-watered seedlings grown from Geohumus grains had longer roots than in the other treatments. 

## 5. Conclusions 

Seedlings grown from seeds coated with either of the two hydro-absorbers, as well as the two sorghum varieties (Piper and Susu) responded differently to fully watered and water deficit conditions. Seeds from both sorghum varieties coated with Stockosorb performed better than those coated with Geohumus in all water treatments. In fully watered conditions, growth in seedlings grown from Geohumus grains was severely reduced. However, unlike in seedlings grown from Stockosorb grains or uncoated seeds, those grown from Geohumus grains had increased leaf growth. Therefore, when analyzing the response of seedlings to drought stress, the type of hydro-absorber coating and variety should be taken into consideration and further research into the specific sugar pathways operating in these sorghum varieties would shed more light in the determination of the most suitable coating materials. In general, promising results for mitigating early season drought effects on seedling establishment were obtained with the coated seeds of both varieties, however, when targeting systems with unreliable rainfall early in the sorghum cropping season in tropical crop production systems, the combination of seed coat constituents and variety needs to be tested carefully to achieve the desired results.

## Figures and Tables

**Table 1:** Second and third leaf elongation rates (mm day^−1^) of Susu and Piper seedlings at different moisture levels. Small and capital letters represents comparison in rates between moisture levels (rows) and comparisons between cereals at the same moisture level for a given day after sowing (DAS) respectively at alpha = 5%. Values in brackets are standard errors.

		Treatments								
		Uncoated			Stockosorb			Geohumus		
					Moisture levels					
	DAS	FC	50% FC	25% FC	FC	50% FC	25% FC	FC	50% FC	25% FC
Variety	Second leaves									
**Susu**	6	6.1 (±1.0) ^aA^	4.7 (±0.6) ^aB^	4.6 (±0.7) ^aA^	5.5 (±0.8) ^aA^	6.3 (±0.8) ^aB^	6.5 (±1.0) ^aA^	7.2 (±0.5) ^aA^	8.4 (±0.9) ^aA^	7.4 (±1.0) ^aA^
	8	3.9 (±0.6) ^aA^	3.1 (±0.3) ^abB^	2.9 (±0.4) ^bB^	3.8 (±0.4) ^aB^	4.7 (±0.4) ^aAB^	3.7 (±0.5) ^aB^	5.2 (±0.4) ^aB^	4.9 (±0.4) ^aA^	6.2 (±0.7) ^aA^
	10	3.3 (±0.5) ^aB^	2.9 (±0.3) ^aB^	2.6 (±0.3) ^aB^	3.3 (±0.4) ^aB^	4.0 (±0.3) ^aA^	3.5 (±0.5) ^aB^	4.5 (±0.4) ^abA^	4.0 (±0.3) ^bA^	5.6 (±0.7) ^aA^
	Third leaves									
	9	26.7 (±2.2) ^aA^	11.3 (±1.3) ^bA^	12.6 (±1.0) ^bAB^	27.6 (±1.8) ^aA^	14.3 (±2.4) ^bA^	13.9 (±1.0) ^bA^	12.0 (±1.3) ^aB^	9.5 (±0.7) ^aA^	10.0 (±1.0) ^aB^
	11	13.2 (±0.8) ^aA^	5.3 (±0.6) ^bB^	5.9 (±0.6) ^bB^	14.1 (±0.9) ^aA^	6.9 (±0.9) ^abB^	7.1 (±0.4) ^bAB^	9.2 (±0.9) ^aB^	10.0 (±0.5) ^aA^	8.8 (±0.8) ^aA^
	13	11.4 (±0.8) ^aA^	6.8 (±0.6) ^bB^	5.9 (±0.6) ^bB^	11.3 (±0.6) ^aA^	7.5 (±0.7) ^abAB^	6.5 (±0.3) ^bAB^	8.1 (±0.6) ^abB^	8.6 (±0.4) ^aA^	7.0 (±0.5) ^bA^
**Piper**	Second Leaves									
	6	15.3 (±1.7) ^aA^	16.4 (±1.1) ^aA^	13.9 (±0.6) ^aA^	6.6 (±0.9) ^aC^	8.7 (±0.7) ^aB^	9.3 (±1.6) ^aB^	10.3 (±1.2) ^aB^	9.8 (±1.0) ^aB^	9.3 (±0.7) ^aB^
	8	8.5 (±0.3) ^aA^	8.2 (±0.4) ^abA^	6.8 (±0.3) ^bA^	5.9 (±0.3) ^aB^	5.1 (±0.4) ^bC^	5.8 (±0.5) ^aA^	6.2 (±0.3) ^aB^	6.6 (±0.8) ^aB^	6.4 (±0.4) ^aA^
	10	8.0 (±0.4) ^aA^	7.2 (±0.4) ^aA^	6.1 (±0.2) ^bA^	4.5 (±0.2) ^aB^	3.7 (±0.3) ^aB^	4.1 (±0.3) ^aA^	4.6 (±0.3) ^aB^	5.0 (±0.7) ^aA^	4.8 (±0.3) ^aA^
	Third leaves									
	9	16.8 (±1.6) ^aA^	13.0 (±1.4) ^abA^	7.9 (±1.3) ^bB^	17.3 (±1.6) ^aA^	12.5 (±2.4) ^aA^	13.3 (±1.9) ^aAB^	13.0 (±1.7) ^aA^	15.8 (±1.3) ^aA^	16.9 (±2.2) ^aA^
	11	17.8 (±1.0) ^aA^	10.2 (±0.9) ^bA^	9.7 (±1.4) ^bA^	12.4 (±0.9) ^aB^	10.4 (±0.9) ^aA^	6.8 (±0.6) ^bA^	10.3 (±0.9) ^aB^	11.0 (±0.8) ^aA^	9.8 (±0.8) ^aA^
	13	14.6 (±0.6) ^aA^	8.7 (±0.6) ^bA^	6.3 (±0.6) ^cA^	9.8 (±0.9) ^aB^	9.5 (±0.7) ^abA^	6.9 (±1.1) ^bA^	9.3 (±1.9) ^aB^	9.5 (±0.7) ^aA^	7.4 (±0.5) ^aA^
